# Characterization of hyperpolarization-activated cyclic nucleotide-gated channels in oligodendrocytes

**DOI:** 10.3389/fncel.2024.1321682

**Published:** 2024-02-26

**Authors:** Kyle A. Lyman, Ye Han, Andrew P. Robinson, Samuel E. Weinberg, Daniel W. Fisher, Robert J. Heuermann, Reagan E. Lyman, Dong Kyu Kim, Andreas Ludwig, Navdeep S. Chandel, Mark D. Does, Stephen D. Miller, Dane M. Chetkovich

**Affiliations:** ^1^Department of Neurology, Massachusetts General Hospital, Boston, MA, United States; ^2^Department of Neurology, Vanderbilt University Medical Center, Nashville, TN, United States; ^3^Department of Microbiology-Immunology and Interdepartmental Immunobiology Center, Northwestern University, Chicago, IL, United States; ^4^Department of Medicine, Northwestern University, Chicago, IL, United States; ^5^Department of Psychiatry, University of Washington, Seattle, WA, United States; ^6^Department of Neurology, Washington University, St. Louis, MO, United States; ^7^Heritage College of Osteopathic Medicine, Ohio University, Dublin, OH, United States; ^8^Department of Biomedical Engineering, Vanderbilt University, Nashville, TN, United States; ^9^Vanderbilt University Institute of Imaging Science, Vanderbilt University, Nashville, TN, United States; ^10^Department of Radiology and Radiological Sciences, Vanderbilt University School of Medicine, Nashville, TN, United States; ^11^Department of Electrical Engineering, Vanderbilt University, Nashville, TN, United States; ^12^Institut fur Experimentelle und Klinische Pharmakologie und Toxikologie, Friedrich-Alexander-Universitat Erlangen-Nurnberg, Erlangen, Germany

**Keywords:** oligodendrocyte, oligodendrocyte progenitor cell, HCN, TRIP8b, EAE, I_*h*_, mitochondria, multiple sclerosis

## Abstract

Mature oligodendrocytes (OLG) are the myelin-forming cells of the central nervous system. Recent work has shown a dynamic role for these cells in the plasticity of neural circuits, leading to a renewed interest in voltage-sensitive currents in OLG. Hyperpolarization-activated cyclic nucleotide-gated (HCN) channels and their respective current (I_*h*_) were recently identified in mature OLG and shown to play a role in regulating myelin length. Here we provide a biochemical and electrophysiological characterization of HCN channels in cells of the oligodendrocyte lineage. We observed that mice with a nonsense mutation in the *Hcn2* gene (*Hcn2^ap/ap^*) have less white matter than their wild type counterparts with fewer OLG and fewer oligodendrocyte progenitor cells (OPCs). *Hcn2^ap/ap^* mice have severe motor impairments, although these deficits were not observed in mice with HCN2 conditionally eliminated only in oligodendrocytes (*Cnp*^*cre*/+^; *Hcn2^F/F^*). However, *Cnp*^*cre*/+^; *Hcn2^F/F^* mice develop motor impairments more rapidly in response to experimental autoimmune encephalomyelitis (EAE). We conclude that HCN2 channels in OLG may play a role in regulating metabolism.

## Introduction

Hyperpolarization-activated cyclic nucleotide-gated channels are encoded by four genes (*Hcn1-4*) in mammals (Notomi and Shigemoto, 2004). These channels mediate a nonspecific cationic current and open at hyperpolarized potentials without inactivating (Wahl-Schott and Biel, 2008). In cardiomyocytes, I_*h*_ plays an important role in rhythmogenesis and in CA1 pyramidal neurons the current regulates excitability and temporal summation (Zolles et al., 2006). HCN channels are subject to multiple levels of regulation and channel opening is influenced by membrane voltage, intracellular cyclic nucleotides, and auxiliary subunit binding (Lyman et al., 2021).

Given the strong link between HCN channels and electrical activity, it is surprising that HCN2 proteins have been observed in mature oligodendrocytes (OLG), the myelin forming cells of the central nervous system (Heuermann et al., 2016; Foote et al., 2019). Mice that are homozygous for a nonsense mutation in the *Hcn2* gene (*apathetic, Hcn2*^ap/ap^**) have a severe behavioral phenotype featuring generalized seizures and severe motor deficits (Chung et al., 2009) identical to genetic knockout of *Hcn2* (Ludwig et al., 2003). These features are reminiscent of human patients with cerebral palsy and raise the possibility of a white matter deficit (Mercimek-Mahmutoglu et al., 2015). Notably, knockout of HCN1 produces a subtle phenotype (Nolan et al., 2004; Santoro et al., 2010) despite being expressed at higher levels in neurons, although not expressed in OLG (Notomi and Shigemoto, 2004).

Unlike oligodendrocyte progenitor cells (OPCs), which are known to receive synaptic input that is thought to guide their differentiation and myelination, mature OLG do not directly receive synaptic contacts from neurons (Paez et al., 2009; Bergles et al., 2010). However, HCN2 channels were recently shown to regulate the length of myelin segments and oligodendrocyte-specific knockout of HCN2, as well as pharmacological blockade of I_*h*_, lead to shorter myelinated segments (Swire et al., 2021). These results point to potential new roles for HCN2 channels beyond those associated with regulating rhythmicity. For example, in both cardiomyocytes and renal cells, HCN channels are expressed in mitochondria and play a role in regulating oxidative phosphorylation (León-Aparicio et al., 2019; Padilla-Flores et al., 2020). In addition to expressing HCN2, mature OLG have also been noted to express high levels of tetratricopeptide repeat-containing Rab8b-interacting protein (TRIP8b) (Zhang et al., 2014). TRIP8b is a variably spliced (Han et al., 2020) auxiliary subunit of HCN channels that has been studied primarily in pyramidal neurons of the cortex and hippocampus (Lyman et al., 2017b; Fisher et al., 2018; Frigerio et al., 2018; Foote et al., 2019). By binding the cytoplasmic domain of HCN subunits, TRIP8b plays a role in regulating surface trafficking and subcellular localization of the channel (Lewis et al., 2011; Han et al., 2017). However, it remains unclear if TRIP8b plays a similar role in OLG.

In this report, we set out to characterize the expression and function of HCN2 channels in the OLG lineage. We found that HCN2 channels are expressed in mature OLGs and that the subcellular distribution of HCN2 is regulated by TRIP8b. *In vitro* studies revealed that HCN2 channels are open at rest and mediate a tonic depolarizing influence on the resting membrane potential of mature OLG. Conditional genetic knockout of HCN2 in OLG leads to a more rapid onset of experimental autoimmune encephalomyelitis (EAE), an animal model of multiple sclerosis (MS). These results suggest that HCN2 may play a role in regulating the metabolism of OLGs and that loss of these channels sensitizes the cells to inflammatory damage.

## Materials and methods

### Animals

All experiments involving animals were performed according to protocols approved by the Institutional Animal Care and Use Committees of Northwestern University and Vanderbilt University Medical Center. Both male and female mice were used for all experiments with the exception of EAE (where only females were used) and rotarod (where only males were used). *Cnp^+/cre^* mice were provided by Dr. Brian Popko (Northwestern University) as a generous gift from Dr. Klaus Armin-Nave (Max Planck Institute of Experimental Medicine, Goettingen, Germany) and genotyped as previously described (Lappe-Siefke et al., 2003). The wild type allele was identified as a 643 bp band as the result of CNP-E3sense 5′-GCCTTCAAACTGTCCATCTC-3′ and CNP-E3antisense 5′-CCCAGCCCTTTTATTACCAC-3′. The CNP-cre allele was detected as a 357 bp fragment using the CNP-E3antisense primer and the puro3 primer 5′-CATAGCCTGAAGAACGAGA-3′.

*Hcn2^F/F^* mice were a generous gift of Dr. Andreas Ludwig (Friedrich-Alexander University, Erlangen, Germany) and genotyped as previously described (Ludwig et al., 2003). The presence of the wild type allele was detected as a 437bp band and the floxed allele as a 488bp allele using the following primers: HCN216F 5′ CAGCTCCCATTTGCCCTTGTGC 3′ and HCN215bR 5′ GGAAAAATGGCTGCTGAGCTGTCT 3′.

The gene encoding TRIP8b is known as *Pex5l*, although for simplicity, we describe it as *Trip8b* in this report. *Trip8b*^–/–^ mice were maintained as previously described (Lewis et al., 2011). The wild type allele was detected as a 150 bp band using TSKC5′ GCCCAATTGATGCATTTACTTTGG 3′ and 1.1b3′ 5′ TGTGCCTATGTCTGCCTTCCCAG. The knockout allele was detected with TSKC5′ as the forward primer and TSKB3′ 5′ CTGGACACAAACTAGAGTCACGG 3′. All oligonucleotides used for genotyping were synthesized by Integrated DNA Technologies (Coralville, IA).

### MRI

Eight mice (4 *Hcn2^+/+^* and 4 *Hcn2^ap/ap^*, with 2 males in each group) were scanned at 7 T with a 25 mm litzcage RF coil. We investigated white matter by evaluating myelin water fraction (MWF) with MRI. In excised mouse brains, this quantity has been histologically correlated to electron microscopy measures of myelin content (West et al., 2018). Four brains were scanned at a time (2 *Hcn2^+/+^* and 2 *Hcn2^ap/ap^*). During each session, brains were loaded into a 3D printed mouse brain holder, bathed in Fomblin and scanned overnight. Each scan consisted of a high-resolution anatomical scan (HRANAT, 50 μm isotropic resolution), and a multiple spin echo (MSE) scan (150 μm isotropic resolution). 3D myelin water fraction (MWF) maps were calculated using MSE scan data [similar to prior work (Grier et al., 2017)]. The HRANAT scan was used to initially register all brain images to a minimum deformation atlas (MDA) from USC LONI Lab. The transformation from these were then applied to the MWF parameter map such that all images and maps are aligned in the same 3D space. MWF of the control mice were compared with those of the knockouts.

### OPC immunopanning

O4^+^ OPCs were isolated by immunopanning and grown in culture as described previously (Rodgers et al., 2015). Male and female C57BL/6J pups (Jackson Labs) were used for experiments involving only wild type cells and in separate experiments, pups from a cross between *Cnp*^+/+^; *Hcn2^F/F^* and *Cnp*^+/*cre*^; *Hcn2^F/F^* animals were used. All pups were aged P7-9 for isolations.

### Electrophysiology

Glass pipettes were pulled using a Sutter P87 pipette puller (2–5 MΩ). All recordings were performed in the whole-cell configuration and were made with a PC-ONE amplifier (Dagan), filtered at 3 kHz, and digitized at 20 kHz using an InstruTECH ITC16. Data analysis was performed using custom written routines in Igor Pro (Lake Oswego, OR) (Han et al., 2017). For extracellular solution, oligodendrocyte media without growth factors or forskolin was used: 463.5 mL DMEM (Invitrogen 11960-069), 5 mL Insulin (0.5 mg/mL Sigma Aldrich), 5 mL Sodium pyruvate (Invitrogen 11360-070), 5 mL Penicillin/Streptomycin (Invitrogen 151340-122), 500 μL Trace Elements B (Invitrogen), 500 μL of Biotin (0.5 g/mL, Sigma Aldrich), 500 μL N-Acetyl-Cysteine (5 mg/mL, Sigma Aldrich), 5 mL SATO (see below), 5 mL glutamine (Invitrogen), and 10 mL of B27 Neurobrew without Vitamin A (Invitrogen). For internal solution (in mM): 130 KCl, 10 NaCl, 0.5 MgCl_2_, 1 EGTA, 5 HEPES, 2 MgATP. SATO was prepared as a 100X stock containing (in 40 mL of Neurobasal media): 400 mg transferrin (Sigma T-1147), 400 mg BSA (Sigma A-4161), 10 μL of 1 mM progesterone (Sigma P8783), 640 μL of 100 mg/mL putrescine (Sigma P-5780), and 400 μL of 50 mg/mL sodium selenite (Sigma S5261). A liquid junction potential of 3 mV was calculated using Clampex (Molecular Devices, San Jose, CA) and was not corrected for.

### Immunohistochemistry

Mice were deeply anesthetized with isoflurane and then transcardially perfused with ice cold phosphate buffered saline (PBS) followed by 4% paraformaldehyde (PFA). The brain was then dissected into a 15 mL conical tube filled with 4% PFA and kept at 4°C for 48–72 h prior to sectioning. Free floating 30 μm coronal sections were generated using a vibratome (Leica, Buffalo Grove, IL). Sections were then preserved at 4°C in PBS supplemented with 0.25% NaN_3_ until staining. Prior to incubation with primary antibody, antigen retrieval was performed using 10 mM sodium citrate buffer (pH 9.0) at 80°C for 10 min. The tissue was then allowed to cool to room temperature for 30 min to 1 h. The sections were then incubated in blocking buffer (PBS with 5% normal goat serum and 0.03% Triton X-100) for 1 h at room temperature with gentle shaking. Primary antibodies were diluted in blocking buffer and the tissue was next incubated overnight at 4°C with gentle shaking. The following day, the sections were washed three times for 5 min each with PBS-T (PBS with 0.03% Triton X-100). Secondary antibodies were applied for 1 h at room temperature in blocking buffer with gentle shaking. The tissue was then washed three times for 5 min in PBS-T. On the final wash, 1 μM DAPI was added to the PBS-T. Sections were then mounted onto microscope slides and allowed to dry overnight at room temperature in the dark. The following day, the slides were coverslipped using PermaFluor (Thermo Fisher Scientific, Fremont, CA) and sealed with clear fingernail polish. Imaging was performed at the Northwestern University Center for Advanced Microscopy on a Nikon confocal microscope using NIS Elements software (Nikon, Melville, NJ).

Primary antibodies for immunohistochemistry: 1:100 mouse anti-CC1 (OP80, Millipore, Temecula, CA), 1:1000 rabbit anti-PDGFRα (a generous gift of Dr. Bill Stallcup), 1:1000 mouse anti-Olig2 (MABN50, Millipore, Temecula, CA), 1:500 rat anti-MBP (MCA409S, Bio-Rad, Hercules, CA), 1:1000 guinea pig anti HCN2 [custom antibody previously validated (Shin et al., 2008; Chung et al., 2009)], and 1:1000 mouse anti-TRIP8b (N212/17, Neuromab, Davis, CA).

Cell counting experiments were performed by selecting every sixth tissue section for quantification. The tissue was then processed for immunohistochemistry as described above. Images were then taken on a confocal microscope by an experimenter blinded to the genotype of the animal. Images were randomized and grids were overlaid onto the image for counting purposes using FIJI. For Figure 3, an average of 6.4 ± 0.4 (mean ± s.e.m.) images per animal were analyzed. For Figure 11, an average of 12.5 ± 0.7 (mean ± s.e.m.) images per animal were analyzed.

### EdU pulse chase labeling

EdU pulse chase labeling was performed using a commercially available kit (Thermo Fisher Scientific, Fremont, CA) following a previously established protocol (Hill et al., 2014). EdU was dissolved in 0.9% normal saline to a concentration of 5 mg/10 mL and frozen at −20°C. On the day of the experiment, aliquots of EdU were thawed and 20 μL/g bodyweight was injected intraperitoneally. A total of 24 h following injection, the mice were deeply anesthetized with isoflurane and perfused for immunohistochemistry as described above. The following modifications to the procedure outlined above were performed to visualize the EdU label. After incubation with the secondary antibody, the sections were blocked for 10 min at room temperature in blocking buffer. The manufacturer’s instructions were then used to conjugate the Alexa Fluor Azide to the EdU label. Afterward the tissue was again incubated for 10 min at room temperature in blocking buffer. Finally, the tissue was washed twice with PBS-T, using 1 μM DAPI in the final wash.

### Immunocytochemistry

Cells grown in culture were washed once using PBS and then fixed for 10 min at room temperature in 4%PFA. The cells were then washed three times in PBS and kept at 4°C in PBS until staining. The coverslips were then blocked for 1 h at room temperature using blocking buffer (see above) prior to an overnight incubation in primary antibodies at 4°C with gentle shaking. The following day the coverslips were washed three times for 5 min in PBS-T, then incubated for 1 h in secondary antibody at room temperature with gentle shaking. Coverslips were then washed three times in PBS-T, with 1 μM DAPI added to the final wash. The coverslips were then mounted onto microscope slides using PermaFluor (Thermo Fisher Scientific, Fremont, CA) and sealed with clear fingernail polish. Primary antibodies used were identical to those described above.

### Western blotting

Western blotting was performed according to a previously described protocol (Lewis et al., 2011; Lyman et al., 2021). Primary antibodies used were custom rabbit anti-HCN2 and commercially available rabbit anti-GAPDH (Santa Cruz Biotechnology, CA). Blots were exposed using a Li-COR Odyssey FC imaging station (Li-COR, Lincoln, NE). Bands were quantified using ImageStudio software and scaled to the GAPDH signal.

### qRT-PCR

Cells grown in 6 well plates were washed once in PBS and then harvested in RNAlater (Qiagen, Valencia, CA) using a cell scraper on ice. Cells were then frozen at −80°C until the next step. RNA was extracted using the PureLink RNA Mini kit (Thermo Fisher Scientific, Fremont, CA) per the manufacturer’s directions and cDNA synthesis was performed using the High Capacity cDNA Reverse Transcription Kit (Thermo Fisher Scientific, Fremont, CA). qRT-PCR reactions were carried out using Power SYBR Green PCR Master Mix (Thermo Fisher Scientific, Fremont, CA). Primers spanning exon-exon junctions were generated for each gene as follows: HCN2-Forward 5′ acttccgcaccggcattgttattg 3′, HCN2-Reverse 5′tcgattcccttctccactatgagg 3′, GAPDH-Forward 5′ tgatgacatcaagaaggtggtgaag 3′, GAPDH-Reverse 5′ tccttggaggccatgtaggccat 3′, CNPase-Forward 5′ ccctctaccttacaacccagc 3′, CNPase-Reverse 5′ ggaccgcttgtcagttgagga 3′, TRIP8b-Forward 5′ TCAAGTTTCACGGTGACCGAACAAG 3′, TRIP8b-Reverse 5′ AGCTCTGGCTGAGATCTGTGTTCTG 3′. Reactions were run in a CFX Connect Real-Time System (Bio-Rad, Hercules, CA) for 40 cycles with a 15 s denaturing step at 95°C followed by a 1 min 56°C annealing and extension step. Melting curves were examined to verify a single product was generated and amplicons were run out on a 2% agarose gel to confirm their identity.

### Rotarod

Male mice were placed on an accelerating Rotarod (Ugo Basile, Italy) that went from 4 to 40 rpm over the course of 5 min. The time to either fall off of the rod or passively complete a rotation was recorded. Each mouse was tested three times per day on three consecutive days for a total of nine trials with 45 min between each trial on a given day.

### Experimental autoimmune encephalomyelitis

Experimental autoimmune encephalomyelitis was induced as previously described (Najm et al., 2015). Briefly, 8- to 10-week-old female *Cnp^+/+^; *Hcn2^F/F^** and *Cnp^+/cre^; *Hcn2^F/F^** mice were subcutaneously injected with 100 μL of an emulsion made up of Complete Freund’s Adjuvant and MOG_35–55_ peptide (Hooke Laboratories). Two injections of 100 ng of pertussis toxin were then given by intraperitoneal injection, the first occurring 1 h after the subcutaneous injection and the second 48 h later. Each day following immunization the mice were scored by the following scale: 1, limp tail; 2, limp tail and hind limb weakness; 3, hind limb paralysis; 4, hindlimb paralysis and forelimb weakness; 5, moribund.

### Flow cytometry

Flow cytometry was performed as previously described (Robinson et al., 2014, 2020) using Millipore anti-O4 (Clone 81, Mab345), Millipore anti-NG2 (Millipore AB5320), and MitoTracker (ThermoFisher M7514) according to the manufacturer’s instruction.

### Statistics

Statistics were calculated using MATLAB (Mathworks, Natick, MA) and electrophysiology data was collected, analyzed, and displayed using Igor Pro (Lake Oswego, OR). All data are presented as means ± SEM in figure captions and in graphs displayed in figures unless otherwise specified. For MRI data, *T*-tests were performed to compare the mean MWF between control and knockout for each mouse brain region. Brain regions showing significant differences in myelin for MWF are highlighted in Figure 2. All remaining statistical tests are reported in the figure legends. The data that support the findings of this study are available from the corresponding author upon reasonable request.

## Results

### *Hcn2^ap/ap^* mice have fewer OPC and OLG

**FIGURE 1:**
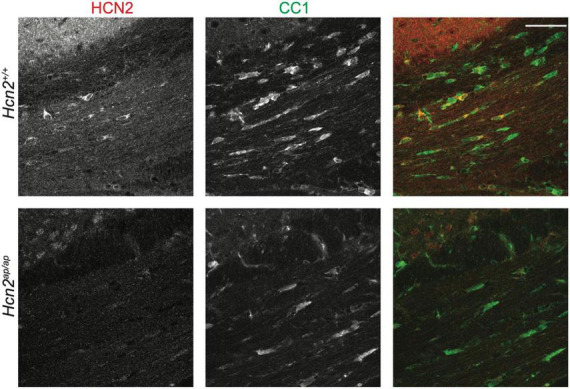
HCN2 is expressed in oligodendrocytes. Immunohistochemistry examining 2 months old *Hcn2^+/+^* and *Hcn2^ap/ap^* littermates to confirm HCN2 expression by CC1+ oligodendrocytes in the corpus callosum. Scale bar is 50 microns.

Previous electron microscopy studies have noted the expression of HCN2 channels at the plasma membrane of mature OLG (Notomi and Shigemoto, 2004). These studies were done without the benefit of a negative control lacking HCN2, so we first sought to confirm these results. Consistent with those findings, we saw high levels of HCN2 expression in OLGs of the corpus callosum [CC1+ cells (Robinson et al., 2014)] in mature mice (Figure 1, see additional images below in Figures 5/6).

**FIGURE 2:**
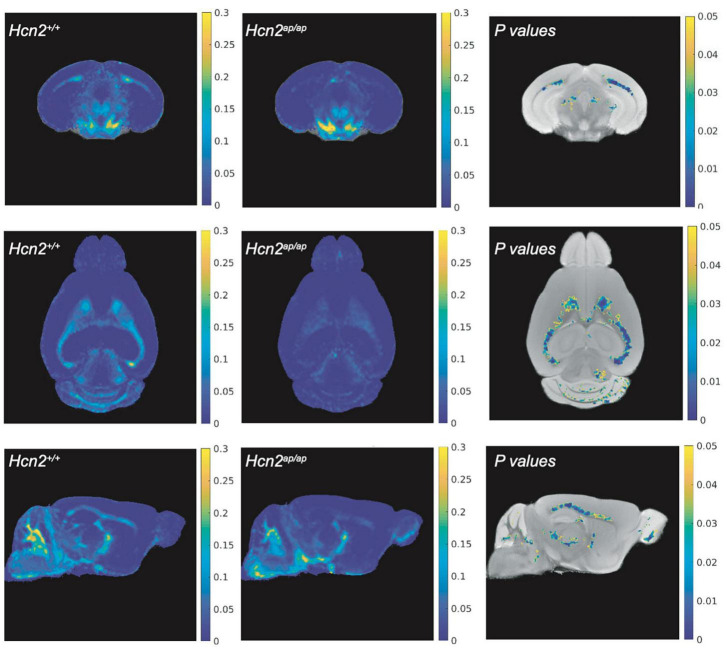
*Hcn2^ap/ap^* mice have less myelin. The brain myelin content of *Hcn2^ap/ap^* mice and wild type littermates was examined using MRI. Each row corresponds to a different view (top: coronal, middle: axial, bottom: sagittal) and each column corresponds to a different condition (left: MWF of *Hcn2^+/+^*, middle: MWF of *Hcn2*^ap/ap^**, right: *p*-values of voxel-by-voxel comparison of *Hcn2^+/+^* and *Hcn2^ap/ap^* MWF maps).

**FIGURE 3:**
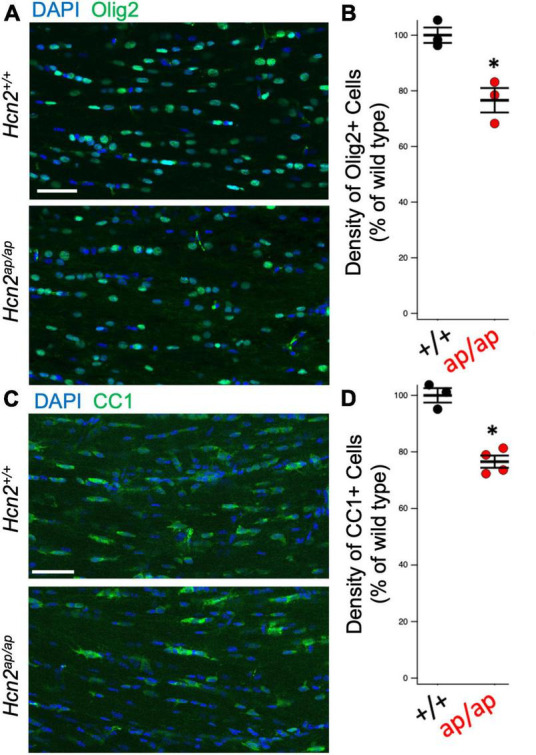
*Hcn2^ap/ap^* mice have fewer oligodendrocytes. **(A)** Representative image of the corpus callosum of P28-P35 animals stained for Olig2, quantified in panel **(B)** (*Hcn2^+/+^* 100.00 ± 2.77; *Hcn2^ap/ap^* 76.64 ± 4.39, *n* = 3, 3, *p* < 0.05, 2 tail T test, mean ± s.e.m. normalized to cells per high-powered field from *Hcn2^+/+^*). **(C)** Immunohistochemistry of the corpus callosum, using CC1 to label mature OLG scale and quantified in panel **(D)**, where there was a significant reduction in CC1+ cells at p28-p35 animals (*Hcn2^+/+^* 100.00 ± 2.55; *Hcn2^ap/ap^* 76.56 ± 2.15, *n* = 3,4, *p* < 0.05, 2 tail T test, mean ± s.e.m. normalized to cells per high-powered-field from *Hcn2^+/+^*). *p < 0.05. Scale bars are 50 microns in panels **(A,C)**.

*Hcn2^ap/ap^* mice are notable for their small size, tremor, motor deficits, and seizures, which could be consistent with a white matter deficit (Ashwal et al., 2004). To investigate this possibility, we performed magnetic resonance imaging (MRI, see *Methods*) to determine brain myelin content. Consistent with our hypothesis, the *Hcn2^ap/ap^* animals showed significantly less myelin in the corpus callosum compared to wild type littermates (Figure 2). We next investigated the density of OLG in *Hcn2^ap/ap^* mice by immunohistochemistry using an Olig2 antibody to label all cells of the OLG lineage (Zhou and Anderson, 2002) and noted a lower density of Olig2+ cells in *Hcn2^ap/ap^* mice at age p28-p35 (Figures 3A, B). We confirmed this result using CC1 to label mature OLG and noted fewer OLG in *Hcn2^ap/ap^* mice (Figures 3C, D).

**FIGURE 4:**
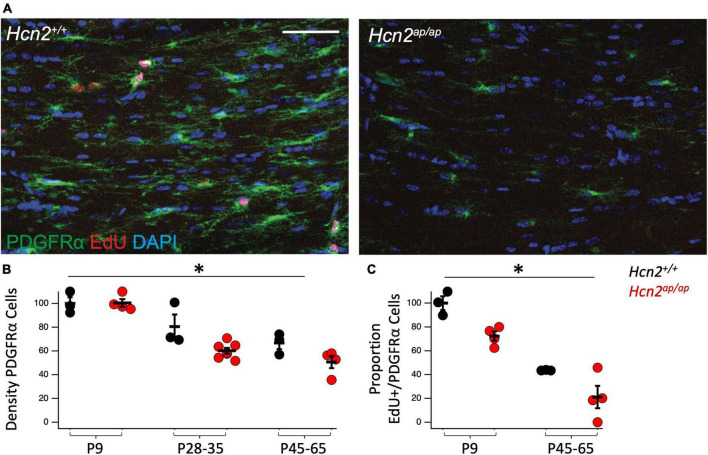
*Hcn2^ap/ap^* mice have fewer proliferating OPCs. **(A)**. Representative images of the corpus callosum of p28-p35 *Hcn2^+/+^* and *Hcn2^ap/ap^* mice. Scale bar is 50 microns. **(B)** Quantification of the density of PDGFRα+ cells, normalized relative to wild type animals at P9: P9 *Hcn2^+/+^* 100.00 ± 5.18, *n* = 3, P28-35 *Hcn2^+/+^* 80.57 ± 10.13, *n* = 3, P45-65 *Hcn2^+/+^* 66.83 ± 5.11, *n* = 3; *Hcn2^ap/ap^* P9 100.42 ± 3.25, *n* = 4, P28-35 *Hcn2^ap/ap^* 60.16 ± 2.49, *n* = 7, P45-65 *Hcn2^ap/ap^* 50.64 ± 5.15, *n* = 4, mean ± s.e.m. There was a significant effect of genotype [*F*(1,18) = 8.71, *p* = 0.008] and an effect of age [*F*(2,18) = 34.65, *p* = 0.00000067], but no interaction between these two terms [*F*(2,18) = 2.41, *p* = 0.11]. **(C)** Quantification of the proportion of PDGFRα+ cells also positive for EdU: P9 *Hcn2^+/+^* 100.00 ± 5.73, P45-65 *Hcn2^+/+^* 43.59 ± 0.16; *Hcn2^ap/ap^* P9 72.34 ± 3.88, P45-65 *Hcn2^ap/ap^* 21.07 ± 9.39. There was a main effect of genotype [*F*(1,10) = 15.01, *p* = 0.0031] and age [*F*(2,10) = 69.13, *p* = 0.00], but no interaction [*F*(2,10) = 0.16, *p* = 0.70]. Note all data is presented scaled to wild type at P9. *Denotes a main effect of genotype in 2 way ANOVA.

Based on the reduction in OLGs, we considered the possibility that there was a deficit in OPC proliferation. Toward this end, we used 5-ethynyl-2’-deoxyuridine (EdU, a thymidine analogue) pulse chase labeling in order to see if there was a difference in the fraction of PDGFRα+ OPCs passing through the cell cycle during a 24 h labeling period (Hill et al., 2014). We noted fewer OPCs in the *Hcn2^ap/ap^* mice as well as a reduction in the proportion of OPCs that were positive for EdU+, establishing a deficit in OPC proliferation (Figure 4).

**FIGURE 5:**
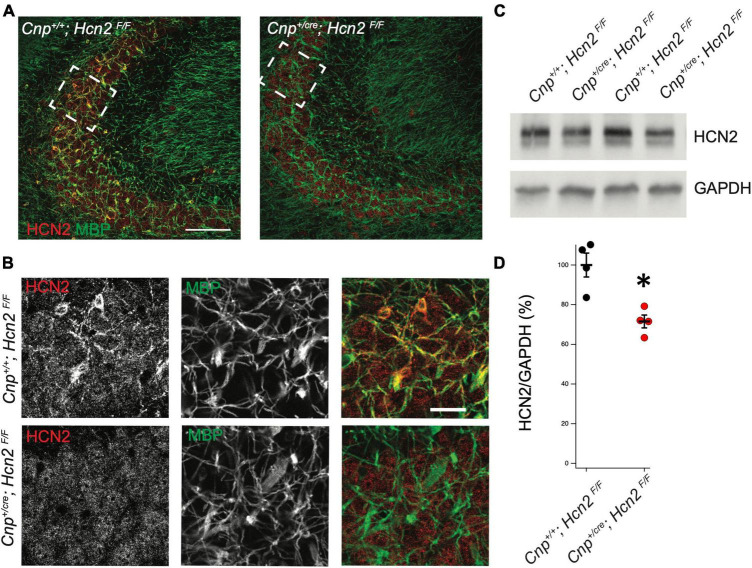
HCN2 is expressed in oligodendrocyte processes. **(A)** Immunohistochemistry was performed to examine expression of HCN2. *Cnp^+/+^;*Hcn2^F/F^** mice express high levels of HCN2 (red) in the MBP positive processes (green). Scale bar represents 100 microns. **(B)** Enlarged area show in brackets in panel **(A)**. Scale bar represents 100 microns. **(C/D)** Whole brain lysate from *Cnp^+/+^;*Hcn2^F/F^** and *Cnp^+/cre^;*Hcn2^F/F^** mice was used for western blotting to examine HCN2 expression normalized to GAPDH expression. Each replicate represents a distinct animal. *Cnp^+/+^;*Hcn2^F/F^** 100 ± 6.01, *n* = 4; *Cnp^+/cre^;*Hcn2^F/F^** 71.53 ± 3.24, *n* = 4, 4, 2 tail t test: *t*(6) = –4.16, *p* = 0.0059, mean ± s.e.m. normalized to *Hcn2^+/+^*. All mice were aged p60-p62. *p < 0.05.

In order to isolate the function of HCN2 in oligodendrocytes, we bred a conditional HCN2 knockout animal by crossing an oligodendrocyte-specific Cre driver (Lappe-Siefke et al., 2003) (2′,3′-cyclic nucleotide phosphodiesterase, *Cnp*^*cre*/+^) with a mouse containing a floxed *Hcn2* allele (*Hcn2^F/F^*) (Ludwig et al., 2003). Upregulation of the *Cnp* gene typically occurs at the OPC stage and is identified as one of the first events of OPC differentiation (Baumann and Pham-Dinh, 2001). Previous work has verified that heterozygosity at the *Cnp* locus does not disrupt OPC maturation either *in vivo* or *in vitro* (Dugas et al., 2010). To verify the level of recombination in oligodendrocytes, we performed immunohistochemistry and observed high levels of HCN2 channel expression in myelin basic protein (MBP) positive cells of *Cnp^+/+^; *Hcn2^F/F^** animals that were absent in *Cnp*^*cre*/+^; *Hcn2^F/F^* littermates (Figures 5A, B). Western blotting using lysate from the cerebrum of mature *Cnp^+/+^; *Hcn2^F/F^** and *Cnp*^*cre*/+^; *Hcn2^F/F^* littermates revealed that 30% of total brain HCN2 is expressed in oligodendrocytes (Figures 5C, D).

### HCN2 expression increases during OPC differentiation

**FIGURE 6:**
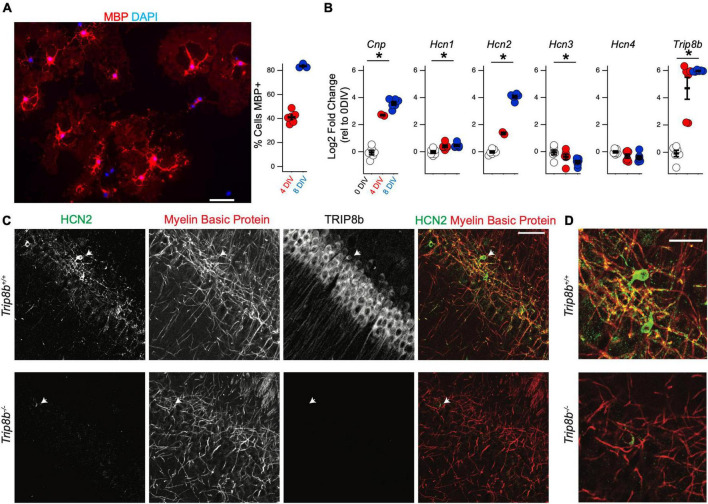
*Hcn2* and *Trip8b* are upregulated during OPC differentiation. **(A)** OPCs were cultured from wild type mice and cultured in differentiation media. Representative culture after 8 days *in vitro* (DIV) is shown, with evidence of myelin basic protein (MBP) expression (red). Scale bar is 50 microns. After 4 DIV an average of 41.09 ± 1.95% (mean ± s.e.m) were positive for MBP and after 8 DIV, an average of 83.61 ± 1.05% (mean ± s.e.m) were positive for MBP. An average of 228.7 ± 26.2 cells were examined from each culture (mean ± s.e.m). **(B)** OPCs were immunopanned from wild type and cells were harvested after 0, 4, or 8 days in differentiation media to examine the effect on RNA expression. All data is presented as log2 fold change relative to 0 days in culture. *Cnp* [0 days –0.04 ± 0.16, 4 days 2.70 ± 0.04, 8 days 3.57 ± 0.13, ANOVA: *F*(2,11) = 158.1, *p* = 7.8 × 10^– 9^, mean ± s.e.m]. *Trip8b* [0 days –0.08 ± 0.23, 4 days 4.69 ± 0.80, 8 days 5.96 ± 0.02, ANOVA: *F*(2,15) = 43.41, *p* = 5.7 × 10^– 7^]. *Hcn1* [0 days –0.01 ± 0.09, 4 days 0.40 ± 0.09, 8 days 0.46 ± 0.06, ANOVA *F*(2,15) = 9.12, *p* = 0.002, mean ± s.e.m]. *Hcn2* [0 days –0.01 ± 0.08, 4 days 1.36 ± 0.08, 8 days 4.06 ± 0.09, ANOVA: *F*(2,11) = 600.11, *p* = 5.8 × 10^– 12^, ANOVA]. *Hcn3* [0 days –0.05 ± 0.18, 4 days –0.37 ± 0.19, 8 days –0.76 ± 0.11, ANOVA *F*(2,15) = 4.54, *p* = 0.02]. *Hcn4* [0 days –0.01 ± 0.07, 4 days –0.31 ± 0.15, 8 days –0.40 ± 0.13, ANOVA *F*(2,15) = 2.72, *p* = 0.09]. *p < 0.05. **(C)** Immunohistochemistry of the cell body layer of CA1 of *Trip8b^+/+^* and *Trip8b*^–/–^ littermates. Scale bar is 50 microns. **(D)** Magnification of cells highlighted with arrow in panel. **(C)** Scale bar is 25 microns.

Previous RNA sequencing efforts have observed a substantial upregulation of HCN2 during OPC differentiation (Zhang et al., 2014). To confirm this result, we next employed the immunopanning technique in order to culture oligodendrocyte lineage cells *in vitro* (Emery and Dugas, 2013). This technique allows for the culture of OPCs with minimal microglial contamination. Under these conditions the cells can be maintained in the OPC stage using media containing platelet derived growth factor (PDGF). Alternatively, by culturing OPCs in the presence of triiodothyronine (T3), these cells are promoted to differentiate into mature OLGs (Emery and Dugas, 2013; Rodgers et al., 2015; Figure 6A).

To examine the developmental regulation of *Hcn2* by the oligodendrocyte lineage, OPCs were collected for qRT-PCR analysis after differentiating for 0, 4, or 8 days in T3 media. RNA expression was quantified using the 2^(-ΔΔC_*T*_) method with *Gapdh* as a reference gene and expression at 0 days in culture as the reference sample (Figure 6B). As expected during OPC differentiation, *Cnp* expression increased with time in culture, and we also noted a substantial increase in the expression of *Hcn2*. In several neuronal subtypes, tetratricopeptide repeat-containing Rab8b-interacting protein (TRIP8b) is an auxiliary subunit of HCN channels responsible for subcellular trafficking of the channels (Lewis et al., 2009; Han et al., 2020); hence we next examined if TRIP8b is also developmentally regulated. Because TRIP8b is extensively alternatively spliced, we used primers amplifying a portion of TRIP8b that is common to all known isoforms. We noted that like *Hcn2, Trip8b* is substantially upregulated during differentiation (Figure 6B). To investigate the significance of this result, we next examined whether or not HCN2 channels expressed in OLG would be influenced by loss of TRIP8b by examining mice lacking all TRIP8b isoforms [*Trip8b*^–/–^ (Lewis et al., 2011)]. Notably, loss of TRIP8b led to a loss of HCN2 channels in the MBP+ processes of mature OLG, indicating that TRIP8b plays a role in scaffolding HCN2 in OLG (Figure 6C).

### Mature OLG express I_*h*_ but not OPCs do not

**FIGURE 7:**
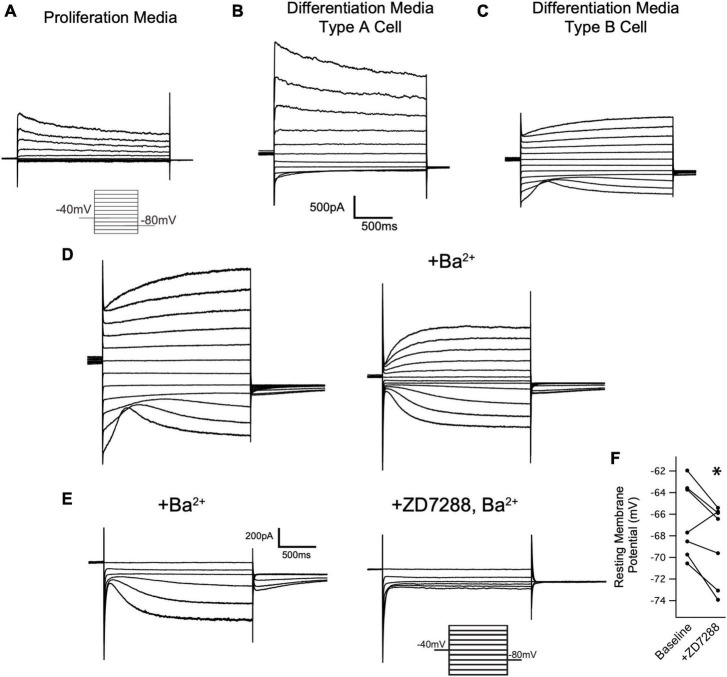
Oligodendrocytes express I_h_. **(A)** Representative recording from an OPC cultured for 24 h in proliferation media after immunopanning. Cells were held at –40 mV and stepped in 20 mV intervals from +60 mV to –140 mV for 2 s at a time. Note the absence of inward currents at hyperpolarized potentials. Caliper scale shown applies to panels **(A–C)**. **(B)** Immunopanned cells maintained in differentiation media led to formation of two cell types that were indistinguishable based on their morphology. Representative ‘Type A’ cell exhibits an inactivating outward current that is activated upon depolarization and a small K_ir_ current, similar to OPCs. **(C)** Representative ‘Type B’ cell. Unlike Type A cells, Type B cells lacked an inactivating current upon depolarization and have a larger K_ir_ current. Additionally, these cells have a second voltage gated conductance that is activated by hyperpolarizing voltage steps. **(D)** Representative mature oligodendrocyte in the absence of extracellular Ba^2+^ (left) and in the presence of 500uM Ba^2+^ (right). Extracellular Ba^2+^ blocks K_ir_ and reveals an inward current that is consistent with I_h_. Caliper scale shown applies to panels **(D,E)**. **(E)** Representative mature oligodendrocyte [distinct from the cell in panel **(D)**] in the presence of 200uM Ba^2+^ (left) and 200uM Ba^2+^ with 20uM ZD7288 (right). The loss of the inward current in the presence of ZD7288 indicates that the current is I_h_. **(F)** To examine if HCN channels are open at resting membrane potentials in mature oligodendrocytes, whole cell recordings were performed in the absence of extracellular Ba^2+^. After recording the membrane potential, 20 μM ZD7288 was applied extracellularly and the change in the membrane potential was noted. [ΔV_m_ = –2.04 ± 0.75mV, *n* = 7, paired t test: *t*(6) = 2.70, *p* = 0.035, mean ± s.e.m.]. *p < 0.05 paired T Test.

We next investigated if I_*h*_ is detectable in cells of the oligodendrocyte lineage. Toward that end, wild type OPCs were cultured and maintained in proliferation media (+PDGF/-T3) for whole cell recordings. Cells were voltage clamped at −40 mV and stepped to a range of test potentials (+60 mV to −140 mV) for 2 s at a time to try and elicit I_*h*_ (if present). Consistent with prior reports on the currents expressed by OPCS *in vitro*, we noted an inactivating current that was apparent upon stepping to depolarized potentials, and a small inwardly rectifying potassium current (K_*ir*_) that was present in some cells (Barres et al., 1988; Soliven et al., 1989; Sontheimer et al., 1996). However, I_*h*_ was not detected in any of these cells (Figure 7A, 0 of 42 cells).

**FIGURE 8:**
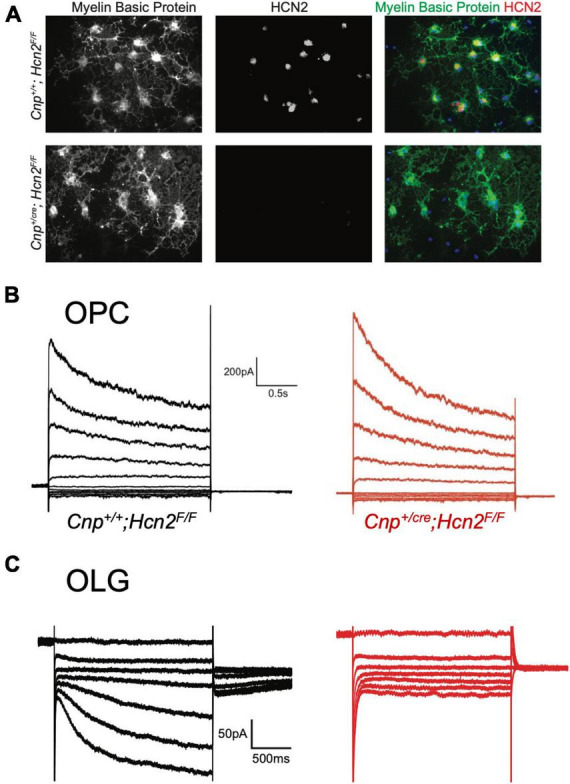
HCN2 mediates I_h_ in oligodendrocytes. **(A)** Wild type and *Cnp^+/cre^;*Hcn2^F/F^** littermates were used for immunopanning and cells were differentiated *in vitro.* After 8 days, the cells were stained for MBP and HCN2. Note the high level of expression of HCN2 in cells that also express high levels of MBP, indicating that more mature oligodendrocytes express HCN2. **(B)** Representative traces from ‘type A’ cells (presumed OPCs) cultured from wild type (*Cnp^+/+^;*Hcn2^F/F^**) and *Cnp^+/cre^;*Hcn2^F/F^** animals. **(C)** Representative traces from wild type and *Cnp^+/cre^;*Hcn2^F/F^** ‘type B’ cells (presumed mature oligodendrocytes) recorded in the presence of 500 μM Ba^2+^. Note the absence of an inward current at hyperpolarized potentials in the *Cnp^+/cre^;*Hcn2^F/F^** cell. In both panels **(B,C)**, the voltage clamp protocol was to hold the cells at –40mV and step to a range of test potentials for 2 s [from +60mV to –160mV in panel **(B)** and from –40mV to –160mV in panel **(C)**], then stepping to –80 mV.

Having noted that both HCN2 and TRIP8b are developmentally upregulated during differentiation, we next asked if HCN2 is present at the surface of mature oligodendrocytes *in vitro* as was recently observed (Swire et al., 2021). Toward that end, OPCs were cultured from wild type animals and then cultured for 6 to 10 days in differentiation media (+T3/-PDGF). After that time, whole cell recordings were made from cells with elaborated (mature appearing) processes. We observed two distinct current profiles in cells recorded under these conditions. First, we noted a cell type with currents that were similar to those expressed by OPCs (Figure 7B) which we refer to as ‘type A cells’. These cells expressed large, inactivating currents that are elicited by depolarization as well as modest K_*ir*_ currents. Given their similarity to the OPCs described above and to the immature oligodendrocytes described in prior reports (Barres et al., 1988; Soliven et al., 1989; Sontheimer et al., 1996), we concluded that these cells were likely immature oligodendrocytes. The second cell type was morphologically indistinguishable from the first but lacked the inactivating current and had a significantly larger K_*ir*_ current. We refer to this second cell type as ‘type B’ cells (Figure 7C, presumptively mature oligodendrocytes). Importantly, in the same voltage regime where the inwardly rectifying channels were active, a second voltage sensitive current was also present. In the presence of extracellular barium to block K_*ir*_, it is clear that the current is I_*h*_ (Figure 7D). The half-activation potential of the current in the presence of extracellular barium was −116.24 ± 7.49 mV (mean ± s.e.m, *n* = 5 cells). The identity of this current was confirmed by its sensitivity to an HCN channel antagonist, ZD7288 [3/3 cells examined, Figure 7E, (Wahl-Schott and Biel, 2008), and the absence of this current in cells cultured from *Cnp*^*cre*/+^; *Hcn2^F/F^* mice, Figure 8].

Based on our own molecular results outlined above, and the similarity to recently published results (Swire et al., 2021), we reasoned that the type B cells were the more mature cells and expressed I_*h*_ while the type A cells were the immature cells that lacked I_*h*_. To confirm that type B cells express I_*h*_ while type A cells do not, we used 200 μM extracellular barium (to block K_*ir*_ and reveal I_*h*_) to determine the fraction of cells of each type that expressed I_*h*_. Consistent with our hypothesis, we noted that 11 of 13 of the type B cells expressed I_*h*_ while 0 of 4 type A cells expressed I_*h*_ (χ^2^= 9.59, *p* < 0.05, see Table 1 for a comparison of membrane parameters). Moreover, at the level of immunocytochemistry, we noted that cells expressing higher levels of MBP also expressed higher levels of HCN2 (Figure 8A).

**TABLE 1:** Comparison of electrophysiological properties of Type A and Type B cells.

	*n*	Membrane capacitance, pF	Membrane resistance, MΩ	Membrane potential, mV
Type A Cells (OPCs)	13	61.83 (7.62)	163.22 (40.25)	−64.19 (2.81)
Type B Cells (OLGs)	19	51.81 (6.64)	135.18 (28.6)	−63.23 (2.85)

*P*-values are shown for two tailed *T* tests. Values are reported as mean (s.e.m.).

We next asked if the HCN channels present in mature oligodendrocytes are open at the resting membrane potential. To answer this question, we performed whole cell recordings from mature oligodendrocytes (identified as Type B cells based on their lack of an inactivating current upon depolarizing voltage steps) and bath applied 20 μM ZD7288 (Magee, 1998, 1999). In response to ZD7288, the resting membrane potential hyperpolarized, indicating a tonic depolarizing influence of I_*h*_ (Figure 7F). These experiments show that mature oligodendrocytes express I_*h*_ and that this current is active at the resting membrane potential.

### I_*h*_ is mediated by HCN2 in mature oligodendrocytes

To determine if any other isoforms of HCN contribute to I_*h*_ in mature oligodendrocytes, OPCs were cultured from both *Cnp^+/+^; *Hcn2^F/F^** and *Cnp*^*cre*/+^; *Hcn2^F/F^* mice and differentiated for 8 days. At the level of immunocytochemistry, it was clear that the recombination rate was nearly 100% (Figures 5A, 8A). I_*h*_ was not detected in any of the Type B *Cnp*^*cre*/+^; *Hcn2^F/F^* cells (0/13), confirming that HCN2 is the only HCN isoform expressed at high levels in mature oligodendrocytes (Figure 8C). However, whole cell recordings did not reveal a difference in membrane capacitance, membrane resistance, or resting membrane potential (Table 2). These results suggest that either the cells homeostatically compensate for loss of HCN2 channel expression or that the cell-to-cell variability in membrane parameters makes a change in these properties difficult to detect.

**TABLE 2:** Comparison of electrophysiological properties of Type B cells from the two genotypes.

Type B cells (OLGs)	*n*	Membrane capacitance, pF	Membrane resistance, MΩ	Membrane potential, mV
*Cnp^+/+^;*Hcn2^F/F^**	15	65.77 (8.49)	186.42 (24.36)	−61.39 (1.94)
*Cnp^+/cre^;*Hcn2^F/F^**	13	68.53 (8.03)	159.31 (27.05)	−59.65 (2.29)

### Loss of HCN2 leads to a reduction in mitochondrial mass in OPCs

**FIGURE 9:**
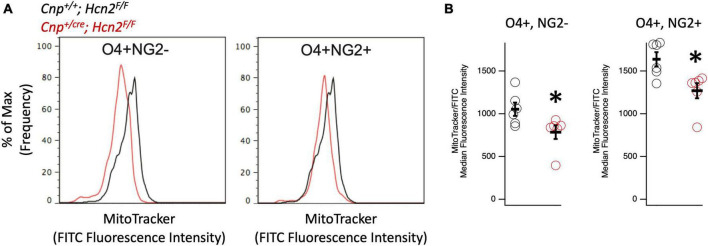
Oligodendrocyte lineage cells from *Cnp*^*cre*/+^; *Hcn2^F/F^* mice have less mitochondrial mass. **(A)** Representative flow cytometry histograms highlighting the reduced MitoTracker signal in both O4+/NG2- cells (left panel) and O4+/NG2+ cells (right panel). **(B)** Quantification of results indicating less mitochondrial mass in O4+/NG2- cells (*Cnp^+/+^; Hcn2*^F/^**^F^ 1053 ± 77.31; *Cnp*^*cre*/+^; *Hcn2^F/F^* 784.83 ± 78.90 *n* = 6,6, 2 tail T test, p<0.05, mean ± s.e.m) and O4+/NG2+ cells (*Cnp^+/+^; Hcn2*^F/^**^F^ 1638.16 ± 83.14; *Cnp*^*cre*/+^; *Hcn2^F/F^* 1270.00 ± 88.31 *n* = 6,6, 2 tail T test, *p* < 0.05, mean ± s.e.m). *p < 0.05.

Given that HCN channels have recently been identified in the mitochondria of several cell types (León-Aparicio et al., 2019; Padilla-Flores et al., 2020), we considered the possibility that loss of HCN2 would negatively affect the mitochondrial mass of oligodendrocyte lineage cells. Immunopanned OPCs from *Cnp^+/+^; *Hcn2^F/F^** cells and *Cnp*^*cre*/+^; *Hcn2^F/F^* cells were incubated in Mitotracker to label mitochondria and subjected to flow cytometry. We examined both O4+/NG2- and O4+/NG2+ oligodendrocyte lineage cells in order to ensure differences in mitochondrial mass weren’t attributable to differences in cell differentiation (Robinson et al., 2014). Both O4+/NG2- and O4+/NG2+ cells from *Cnp*^*cre*/+^; *Hcn2^F/F^* mice had less mitochondrial mass compared with wild type controls (Figure 9).

### Conditional knockout of HCN2 does not affect the oligodendrocyte lineage at baseline

**FIGURE 10:**
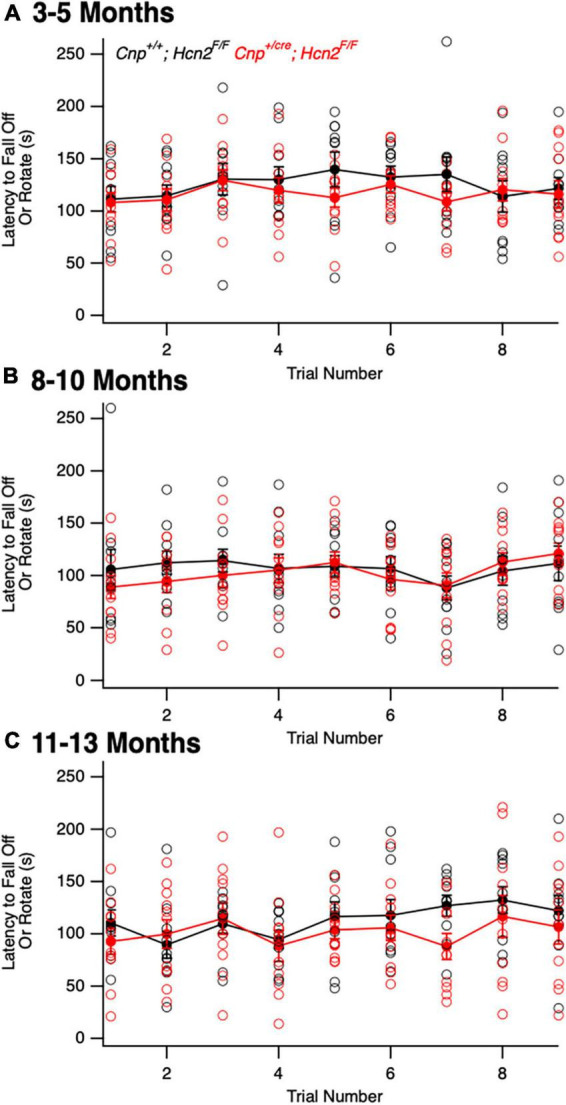
*Cnp*^*cre*/+^; *Hcn2^F/F^* mice do not exhibit a baseline motor deficit. A cohort of *Cnp^+/+^;*Hcn2^F/F^** and *Cnp^+/cre^;*Hcn2^F/F^** mice were allowed to mature for 3–5 **(A)**, 8–10 **(B)**, and 11–13 **(C)** months. No difference in rotarod performance was noted at any time point. **(A)** Age: *Cnp^+/+^;*Hcn2^F/F^** 146 ± 21.9 days, median ± s.d., *Cnp^+/cre^;*Hcn2^F/F^** 149 ± 21.5 days, *n* = 11, 11. A repeated measures ANOVA did not detect a difference between the two genotypes [*F*(8,160) = 0.68, *p* = 0.70]. **(B)** Age: *Cnp^+/+^;*Hcn2^F/F^** 265 ± 20.9 days, median ± s.d., *Cnp^+/cre^;*Hcn2^F/F^** 280 ± 21.5 days, *n* = 10, 11. A repeated measures ANOVA did not detect a difference between the two genotypes [*F*(8,152) = 0.68, *p* = 0.7]. **(C)** Age: *Cnp^+/+^;*Hcn2^F/F^** 350 ± 20.9 days, median ± s.d., *Cnp^+/cre^;*Hcn2^F/F^** 365 ± 21.5 days, *n* = 10, 11. A repeated measures ANOVA still did not detect a difference [*F*(8,152) = 0.81, *p* = 0.59].

**FIGURE 11:**
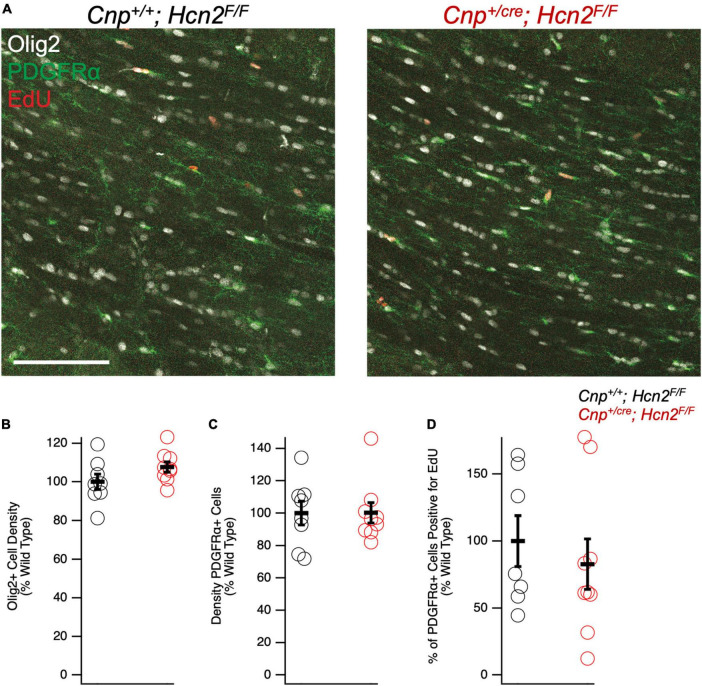
*Cnp*^*cre*/+^; *Hcn2^F/F^* mice have no difference in oligodendrocyte lineage cell density. **(A)** Representative images of the corpus callosum from p28-p35 wild type and *Cnp*^*cre*/+^; *Hcn2^F/F^* mice. Scale bar indicates 100 microns. **(B–D)** Quantification of Olig2+ cell density (*Cnp^+/+^;*Hcn2^F/F^** 100.00 ± 4.01; *Cnp^+/cre^;*Hcn2^F/F^** 107.65 ± 2.64, 2 tail T test, *p* > 0.05, *n* = 8,9), PDGFRα+ cell density(*Cnp^+/+^;*Hcn2^F/F^** 100.00 ± 7.28; *Cnp^+/cre^;*Hcn2^F/F^** 100.19 ± 6.27, mean ± s.e.m., *n* = 8,9, 2 tail T test, *p* > 0.05), and fraction of PDGFRα+ cells that are positive for EdU (*Cnp^+/+^;*Hcn2^F/F^** 100.00 ± 18.97; *Cnp^+/cre^;*Hcn2^F/F^** 82.79 ± 18.88, 2 tail T test, *p* > 0.05, *n* = 7,9). Data in panels **(B–D)** show individual datapoints (corresponding to individual mice, open circles) with mean ± s.e.m superimposed. Values are presented as cells per high powered field scaled to *Cnp^+/+^;*Hcn2^F/F^**.

We next turned our attention to the possibility of an oligodendrocyte phenotype *in vivo.* Unlike *Hcn2^ap/ap^* mice, *Cnp^+/cre^;*Hcn2^F/F^** mice did not exhibit a gross behavioral phenotype and were indistinguishable by eye from *Cnp^+/+^;*Hcn2^F/F^** littermates. To determine if there were subtle deficits in motor coordination, we performed repeated rotarod testing on a cohort of male mice aged 3–5 months, 8–10 months, and 11–13 months, but found no differences (Figure 10). Despite the lack of a motor phenotype, we reasoned that there could still be a deficit in oligodendrocyte density. We next performed EdU pulse-chase labeling, as was performed above in *Hcn2^ap/ap^* animals. There was no difference in either the density of OPCs (identified as PDGFRα+ cells, Figures 11A/C) in the corpus callosum nor in the proportion of double positive PDGFRα+/EdU+ cells (Figure 11D). To examine other stages of the oligodendrocyte lineage, we also stained for Olig2+ cells, but again no difference was noted (Figure 11B). These results indicate that in contrast to the *Hcn2^ap/ap^* animals, the conditional knockout animals do not exhibit a gross deficit in oligodendrocyte density or OPC proliferation.

### *Cnp^+/cre^;*Hcn2^F/F^** mice have a more rapid onset of EAE

**FIGURE 12:**
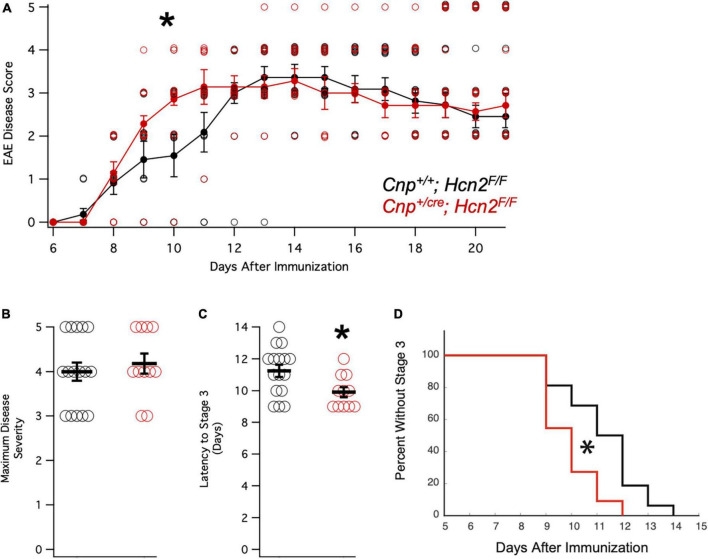
*Cnp*^*cre*/+^; *Hcn2^F/F^* mice develop motor deficits in EAE more rapidly. **(A)** Plot of mean disease severity score versus time for *Cnp^+/+^; *Hcn2^F/F^** and *Cnp*^*cre*/+^; *Hcn2^F/F^* mice. A repeated measures ANOVA revealed a main effect of time [*F*(10,250) = 58.817, *p* = 5.7334e-60] and an interaction of time with genotype [*F*(10,250) = 1.9007, *p* = 0.045]. *Denotes significant difference by ANOVA. **(B)** No difference in maximum disease severity was noted between the two groups [Maximum disease score: *Cnp^+/+^;*Hcn2^F/F^** 4.00 ± 0.20; *Cnp^+/cre^;*Hcn2^F/F^** 4.18 ± 0.22, *n* = 11,16, *t*(25) = −.587, *p* = 0.56, mean ± s.e.m]. **(C)** *Cnp*^*cre*/+^; *Hcn2^F/F^* mice reached stage 3 of disease severity faster than *Cnp^+/+^; *Hcn2^F/F^** [Latency to stage 3: *Cnp^+/+^;*Hcn2^F/F^** 11.25 ± 0.38 days; *Cnp^+/cre^;*Hcn2^F/F^** 9.90 ± 0.31, *t*(25) = 2.52, *p* = 0.018]. *p < 0.05 by two tailed T test. **(D)** Kaplan-Meier curve demonstrating the latency to stage 3. *p < 0.05 by log-rank test.

Having observed a reduction in mitochondrial mass *in vitro* without a deficit in oligodendrocyte density *in vivo*, we considered the possibility that oligodendrocytes from *Cnp^+/cre^;*Hcn2^F/F^** mice might only have a phenotype in the context of stress. We next immunized a cohort of female mice with MOG_35–55_ peptide for experimental autoimmune encephalomyelitis (EAE), a model of multiple sclerosis (Figure 12A). Interestingly, while we did not observe a difference in the severity of the disease (Figure 12B), we noted that the conditional knockout animals developed disease more rapidly (Figures 12C/D). These results suggest that the loss of HCN2 in oligodendrocytes hastens the onset of symptoms in EAE without affecting the severity of the disease.

## Discussion

### HCN2 channels mediate I_h_ in oligodendrocytes

We observed that HCN2 channels are upregulated during OPC differentiation *in vitro* into mature OLG where the channels are active at the resting membrane potential and contribute a depolarizing influence. Our results are consistent with a recent report that similarly described HCN2 channels active at the resting membrane potential of mature OLG cultured from rats (Swire et al., 2021). Although we did not detect I_h_ in cultured OPCs, whole cell recordings made from NG2+ cells of the hippocampus have indeed shown the presence of a current suggestive of I_h_ (Bergles et al., 2000; Lin and Bergles, 2004; Larson et al., 2016). These results raise the possibility that the electrophysiological profile *in vivo* differs from that *in vitro.*

### The function of HCN2 in oligodendrocyte lineage cells

Despite the severe motor phenotype of *Hcn2^ap/ap^* animals, conditional knockout of HCN2 only in cells of the oligodendrocyte lineage did not produce a difference in the number of oligodendrocyte lineage cells. However, despite the lack of a baseline difference in motor function between wild type and conditional knockout mice (*Cnp^+/+^;*Hcn2^F/F^** and *Cnp^+/cre^;*Hcn2^F/F^**, respectively) on rotarod, the conditional knockout mice developed deficits more rapidly during EAE. While there are multiple possible explanations for these findings, an intriguing hypothesis is that HCN2 plays a role in regulating the metabolism of oligodendrocytes. Mitochondrial insults have been repeatedly implicated in the neurological deficits of multiple sclerosis (Mao and Reddy, 2010; Sadeghian et al., 2016), and we observed a reduction in mitochondrial mass of oligodendrocyte lineage cells from *Cnp^+/cre^;*Hcn2^F/F^** animals. How the loss of HCN2 in oligodendrocyte lineage cells is related to the reduction in mitochondrial mass is unclear, although recent work has shown that HCN channels are expressed in the mitochondria of cardiomyocytes and renal cells (León-Aparicio et al., 2019; Padilla-Flores et al., 2020). In those reports, HCN channel function was shown to facilitate K^+^ entry into mitochondria and ultimately increase ATP synthesis via oxidative phosphorylation (León-Aparicio et al., 2019; Padilla-Flores et al., 2020). Extrapolating from these results, loss of HCN2 from OLG mitochondria would limit ATP production, which could explain the sensitization of *Cnp^+/cre^;*Hcn2^F/F^** animals to EAE. However, electron microscopy studies of HCN2 in oligodendrocytes have not revealed the channel in mitochondria (Notomi and Shigemoto, 2004), so more work is needed to clarify the relationship between HCN2, oligodendrocyte metabolism, and susceptibility to EAE. Regardless as to how the deficit in mitochondria comes about, the observed reduction in mitochondrial mass represents a potential liability during times of stress (Kalman et al., 2007).

### TRIP8b regulates HCN2 expression in oligodendrocytes

TRIP8b is subject to extensive splicing, although the function of the majority of the splice isoforms have yet to be studied *in vivo* (Lewis et al., 2009; Han et al., 2020). We observed a substantial increase in TRIP8b expression during oligodendrocyte differentiation, consistent with prior sequencing results (Zhang et al., 2014), but we did not clarify which isoforms were specifically upregulated. A handful of isoforms derived from the ‘1a’ promoter are responsible for subcellular trafficking of HCN channels in CA1 pyramidal neurons, and one report identified expression of isoforms derived from the ‘1b’ promoter in oligodendrocytes (Piskorowski et al., 2011). *In vitro* work suggests that some isoforms derived from the ‘1b’ promoter may play a role in shuttling HCN channels from the cell surface to intracellular compartments, although there is limited *in vivo* data on the function of these ‘1b’ promoter derived isoforms. We have established that TRIP8b plays a role in trafficking HCN2 channels in mature oligodendrocytes, a role that is analogous to its function in CA1 pyramidal neurons (Lewis et al., 2011; Lyman et al., 2021). Unlike the hippocampus, where the role of dendritic HCN channels has been well established (Magee, 1998, 1999), the importance of the subcellular distribution of HCN2 in oligodendrocytes remains opaque. Knockout of HCN2 limits myelin sheath length (Swire et al., 2021), and an intriguing, but untested, possibility is that I_h_ sculpts the OLG response to nearby neuronal signaling to influence myelination (Paez et al., 2009; Gibson et al., 2014).

### *Hcn2^ap/ap^* mice have significant white matter deficits

*Hcn2^ap/ap^* mice have a severe phenotype reminiscent of cerebral palsy in humans while the behavioral deficits of *Cnp^+/cre^;*Hcn2^F/F^** mice are only apparent in EAE. This raises the possibility that the *Hcn2^ap/ap^* phenotype is the result of loss of HCN2 in neurons, but it is also possible that a different Cre driver that eliminates HCN2 earlier in the oligodendrocyte lineage would reproduce the *Hcn2^ap/ap^* phenotype. *Cnp* mediated recombination occurs early in the process of differentiation and we did not observe I_h_ in cultured OPCs (Baumann and Pham-Dinh, 2001). These results suggest that the absence of surface HCN2 channels in OPCs does not preclude differentiation or maturation into functional oligodendrocytes. Despite high expression levels, and the presence of subcellular regulation by TRIP8b, the precise function of HCN2 channels in oligodendrocyte lineage cells remains unknown. It is notable that loss of HCN2 in oligodendrocytes was not associated with an overt behavioral phenotype in the absence of EAE, suggesting the therapies directed at HCN channels [as has been suggested for the treatment of Major Depressive Disorder and delirium (Han et al., 2015, 2022; Lyman et al., 2017a; Lyman, 2023)] are unlikely to cause adverse effects on oligodendrocytes.

## Data availability statement

The raw data supporting the conclusions of this article will be made available by the authors, without undue reservation.

## Ethics statement

The animal study was approved by the Institutional Animal Care and Use Committees of Northwestern University and Vanderbilt University Medical Center. The study was conducted in accordance with the local legislation and institutional requirements.

## Author contributions

KL: Conceptualization, Investigation, Methodology, Writing—original draft, Writing—review and editing. YH: Conceptualization, Investigation, Methodology, Writing—original draft, Writing—review and editing. AR: Investigation, Methodology, Writing—review and editing. SW: Investigation, Methodology, Writing—review and editing. DF: Investigation, Methodology, Writing—review and editing. RH: Investigation, Methodology, Writing—review and editing. RL: Investigation, Methodology, Writing—review and editing. DK: Investigation, Methodology, Writing—review and editing. AL: Investigation, Methodology, Writing—review and editing. NC: Investigation, Methodology, Writing—review and editing. MD: Investigation, Methodology, Writing—review and editing. SM: Investigation, Methodology, Writing—review and editing. DC: Investigation, Methodology, Writing—original draft, Writing—review and editing.
